# Enhanced Cell Viability and Migration of Primary Bovine Annular Fibrosus Fibroblast-like Cells Induced by Microsecond Pulsed Electric Field Exposure

**DOI:** 10.1021/acsomega.3c03518

**Published:** 2023-09-30

**Authors:** Prince M. Atsu, Connor Mowen, Gary L. Thompson

**Affiliations:** † Department of Chemical Engineering, Rowan University, Glassboro, New Jersey 08028, United States; ‡ Department of Biomedical Engineering, Rowan University, Glassboro, New Jersey 08028, United States

## Abstract

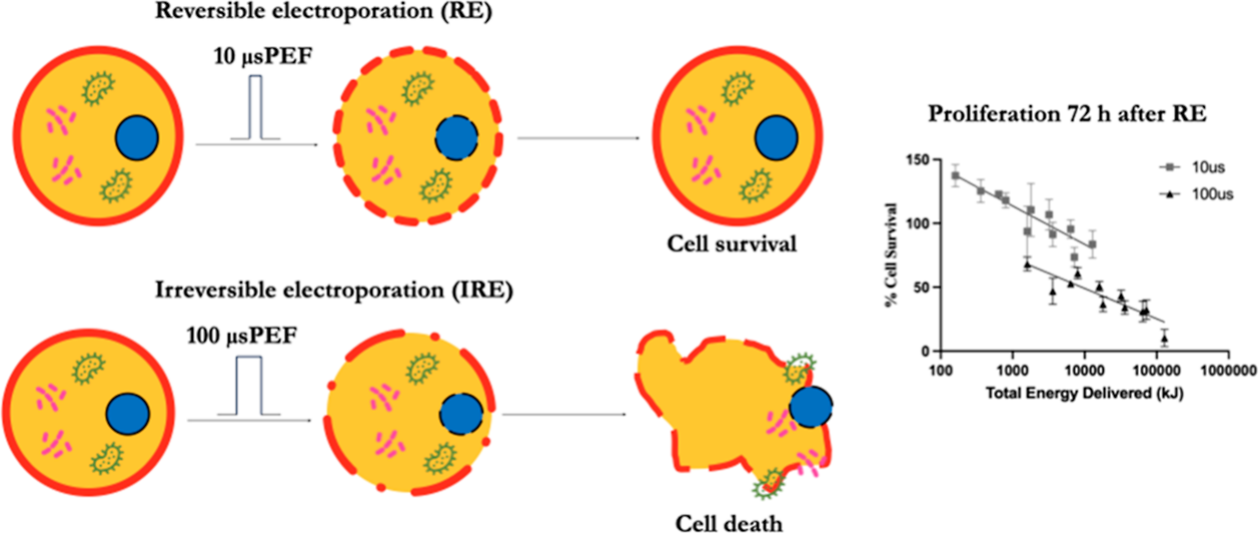

This study is the first to report the enhancement of cell migration and proliferation induced by in vitro microsecond pulsed electric field (μsPEF) exposure of primary bovine annulus fibrosus (AF) fibroblast-like cells. AF primary cells isolated from fresh bovine intervertebral disks (IVDs) are exposed to 10 and 100 μsPEFs with different numbers of pulses and applied electric field strengths. The results indicate that 10 μs-duration pulses induce reversible electroporation, while 100 μs pulses induce irreversible electroporation of the cells. Additionally, μsPEF exposure increased AF cell proliferation up to 150% while increasing the average migration speed by 0.08 μm/min over 24 h. The findings suggest that the effects of PEF exposure on cells are multifactorial—depending on the duration, intensity, and number of pulses used in the stimulation. This highlights the importance of optimizing the μsPEF parameters for specific cell types and applications. For instance, if the goal is to induce cell death for cancer treatment, then high numbers of pulses can be used to maximize the lethal effects. On the other hand, if the goal is to enhance cell proliferation, a combination of the number of pulses and the applied electric field strength can be tuned to achieve the desired outcome. The information gleaned from this study can be applied in the future to in vitro cell culture expansion and tissue regeneration.

## 1 Introduction

Cartilage tissue has limited repair capabilities during injuries. Cartilage heals poorly or does not heal at all, leading to tissue degradation. The cells in injured cartilage become senescent, releasing chemicals that cause inflammation.^1,2^ Cell therapy holds great potential in addressing cartilage degradation; however, several challenges remain for clinical translation. Some of the challenges with cell therapy include appropriate maintenance of cell state, expanding cells reproducibly in large quantity for transplantation, assuring efficient differentiation into desired cell types, and maintaining cell viability and migration during and after delivery.^3–7^ Cellular responses such as proliferation and differentiation can be induced and controlled by physical methods such as mechanical and electrical stimulations and chemical methods such as substrate and material design.^8^ Electric fields have been used to induce cellular phenomena by modifying the membrane potential via voltage-gated channel activity and increased ion transport.^9^ Electrical stimulation-based therapies require minimization of side effects and tunability. For optimization, the treatment time of electric field stimulation can be shortened to ultrashort duration pulsed electric fields (PEF) at higher voltages without permanently damaging cells.^10,11^

The several different cellular responses to PEF have the potential to be utilized in tissue engineering and regenerative medicine. The parameters of PEFs can be chosen such that temporary or permanent pores can be created in the cell membrane. It has been observed that different cell types respond differently to PEF. Therefore, optimization of pulse parameters for electropermeabilization must be cell type or cell line specific.^12,13^ Cellular responses to PEF exposure are multifactorial and initially depend on the dielectric nature of the plasma membrane. The effects of PEF exposures are functions of the applied field intensity, pulse duration or width, the number of pulses, and the pulse repetition rate.^8,14^ The optimal pulse parameters also are influenced by the electrosensitivity of the cells within a threshold PEF exposure proximity, which includes properties such as the cell radius and the type of tissue.^12,13,15^

PEF can be delivered with different pulse durations, including milliseconds (ms), microseconds (μs), and nanoseconds (ns). Each of these pulse durations can have different effects on cells.^16–18^ The duration of the pulses used in the PEF can affect the efficiency and effectiveness of the treatment. Longer pulse durations result in larger electropores within the plasma membrane, which can result in lysis and cell death.^19^ Shorter pulse durations, on the other hand, are less effective at inducing electropores large enough to result in lysis but instead generate greater numbers of smaller sized electropores that still can lead to changes in cell behavior, such as increased permeability or altered gene expression.^20^

Cytotoxic effects of PEF have been extensively studied in several cell lines. However, there is no consensus whether any single exposure metric can reliably and universally predict cell death due to PEF exposure. Ultimately, the choice of pulse duration in the PEF depends on the specific application and desired outcome. For instance, Ibey et al. measure the absorbed dose-dependent survival of Jurkat and U937 cell lines with trains of electric pulses to find that μsPEF exposure causes indiscriminate cell death, whereas nsPEF exposure selectively causes cell death.^21^ Pakhomov et al. determine that the survival of U937 cells following nsPEF exposure depends strongly on pulse duration.^10^ Cemazar et al. observe that the electrosensitivity of different cell lines depends on the type of tissue.^12^ Perhaps, most relevant is the study by May et al.,^22^ who examine the transfection efficiencies of bovine AF cells using millisecond PEF exposures. However, they report neither the electric field strengths of exposures nor the responses of nontransfected AF cells to PEF exposure. AF cell survival, proliferation, and motility in response to μsPEF exposure have not been studied. Although the AF tissue environment is different from AF cells cultured in media,^23^ in vitro investigations of cellular responses induced by PEF exposures serve as a guide for in vivo applications such as tissue regeneration.

PEF parameters can be chosen such that not only cell death can be achieved but also other cellular activities can be enhanced. There have been studies conducted on the effects of external electric fields on proliferation of different cells and cell lines.^9,24–26^ One of the main mechanisms by which μsPEFs influence cell proliferation is through the induction of transient membrane permeabilization or electroporation. Physical phenomena can influence cell proliferation through the activation of intracellular signaling pathways,^27^ such as the mitogen-activated protein kinase pathway, the phosphatidylinositol 3-kinase (PI3K)/Akt pathway, and the Janus kinase/signal transducer and activator of transcription pathway.^28–30^ These signaling pathways can regulate cell proliferation by promoting cell cycle progression and inhibiting cell death.

Electric fields have also been observed to affect the migration of cells,^31–33^ differentiation,^34,35^ and increase of growth factor and DNA syntheses.^36^ Cell migration is a complex process that involves the coordinated movement of cells in response to various signals, such as chemical gradients, mechanical cues, and electrical fields.^37,38^ One of the main mechanisms by which μsPEFs affect cell migration is through the modulation of the cytoskeletal dynamics. These changes can influence cell adhesion, traction, and contractility, which are critical for cell migration.^39,40^

Cell proliferation and migration play significant roles in tissue regeneration. Slow proliferation significantly impedes the regeneration of tissue.^9^ For this reason, PEF-induced AFC proliferation has the potential to enhance the regeneration of the AF tissue. Similarly, cell migration is vital to mammalian cell embryonic growth;^41^ hence, systematic PEF-induced AFC migration can be valuable to AF tissue regeneration.

In this study, we characterize the effects of microsecond-duration PEF (μsPEF) on AF cells in vitro to determine the lethal dose parameters and a relevant combination of electric field parameters to enhance cell proliferation and migration. Our findings demonstrate that 10 μsPEF exposures have the potential of enhancing AF cell proliferation with viability increasing up to 150% and enhanced migration speed up to 0.08 μm/min.

## 2 Results

The experimental design investigates the electroporation effect of μsPEF exposure of primary AF cells isolated from bovine AF tissue. The viability of the cells at 24 and 72 h after exposure to various combinations of applied field strength and number of pulses of 10 and 100 μsPEF has been determined using the MTT cell viability assay. The effect of μsPEF exposure on cell migration was measured for adherent AF cells in culture using a scratch wound healing method.

### 2.1 Viability at 24 and 72 h Postexposure

**Figure 1:**
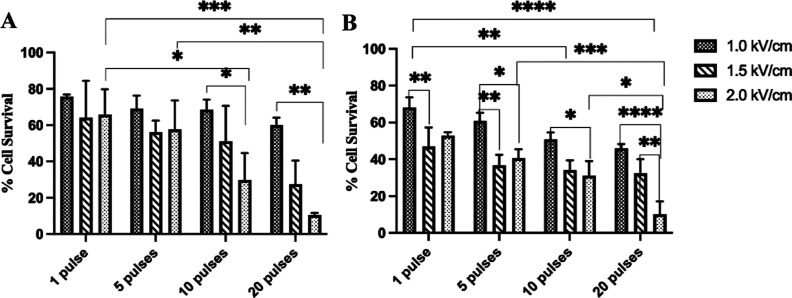
Cell viability after exposure to 100 μsPEF with varying numbers of pulses and electric field intensities. (A) AF cell viability at 24 h postexposure. (B) AF cell viability at 72 h postexposure. Data are from *n* = 5 independent experiments and represent mean values with error bars of 1 standard deviation (SD). Statistical significance is represented by *p* < 0.0001, and for comparison, the number of asterisks (*) represent the decimal places of the *p* value.

**Figure 2:**
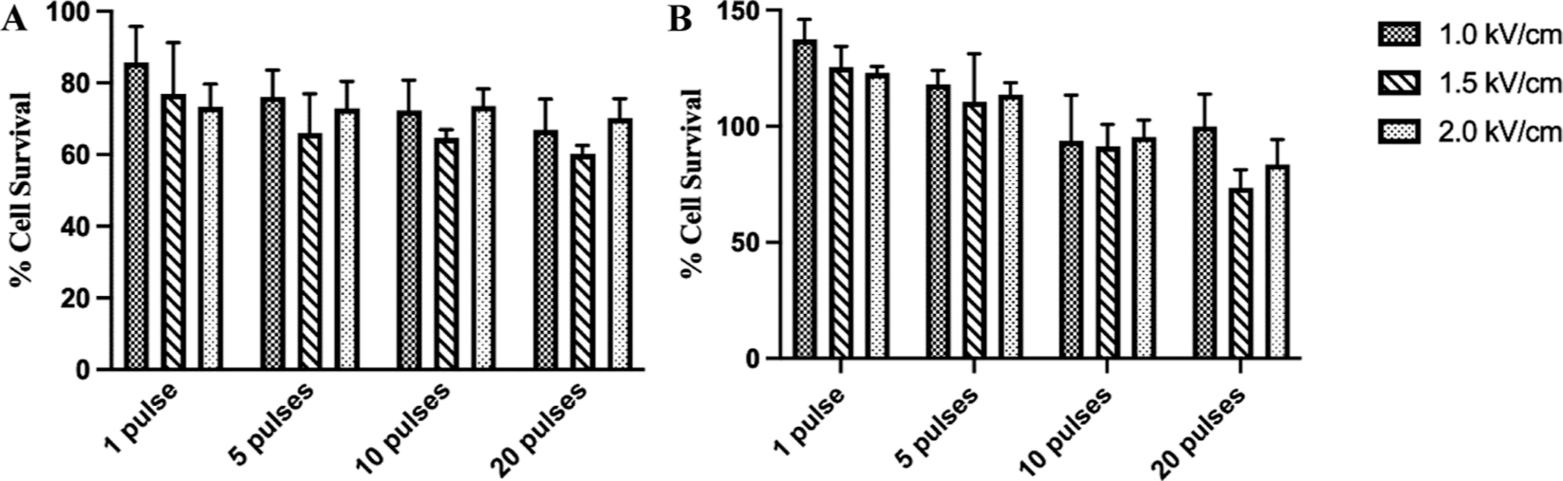
Mean values of AF cell viability after exposure to 10 μsPEF. (A) AF cell viability at 24 h postexposure. (B) AF cell viability at 72 h postexposure. Cell viability exceeds 100% of the controls’ mean values, and hence, the *y*-axis goes up to 150%. Data are from *n* = 5 independent experiments, and error bars represent 1 SD. No statistically significant differences in mean AF cell viability are found among these different exposures to 10 μsPEF when using *p* < 0.001.

Viability results are listed in Figures 1 and 2. The viability is reported as a percentage of the sham controls. After 24 h, the viability of PEF-exposed cells decreases (Figure 1A) for cells exposed to the 100 μs pulse width and all pulse parameters. Increasing the number of pulses generally decreases cell viability after 24 h for a 100 μs pulse width. However, no linear relationship is observed with the applied electric field strength. At a lower number of pulses (1 and 5), there is no statistical difference between the electric field strength effects, whereas the impact of field strength becomes more significant with a higher number of pulses (10 and 20). For 100 μsPEF, AF cells experience further decrease in viability between 24 and 72 h after exposure to 1 or 5 pulses (Figure 1B).

Figure 2 shows the viability of primary AF cells 24 and 72 h after PEF exposure to a pulse of 10 μs duration. The cell viability decreases 24 h postexposure (Figure 2A). The fraction of the cell population experiencing cell death increases with increasing number of pulses. Yet at 72 h postexposure, the percentage of viable cells increases (Figure 2B). Given a smaller number of 10 μs pulses (1 or 5), exposed cell samples even proliferate on average faster than sham control samples. However, given 10 or 20 pulses, the cell proliferation is not as significant at all applied field strengths. When compared to cells treated with 100 μsPEF (Figure 1B), the 10 μsPEF-treated cells have a significant rate of survival at 72 h post exposure, even given 10 or 20 pulses (Figure 2B).

Statistical analysis shows that the observed cellular responses to both the 10 and 100 μs pulse widths are strongly influenced by the number of pulses. The ratio of the *p*-values for a given number of pulses for the PEF exposure with 10 μs pulse width to 100 μs pulse width is greater than 200. This shows the extreme dependence of the observed effect of the 100 μs pulse width on the number of pulses compared to that of the 10 μs pulse width. On the other hand, the applied electric field strength only dominates the cell responses observed for the 100 μsPEF exposures with a *p*-value < 0.0001. Though the effect of the applied electric field strength is not significant for the 24 h postexposure analysis of the 10 μs pulses, the 72 h postexposure analysis shows a significant dependence on the applied electric field strength with a *p*-value = 0.025. Therefore, the cell viability reductions observed 24 h after μsPEF exposures can be attributed mainly to the number of pulses, while enhanced proliferation and cell viability changes at 72 h can be attributed to a combination of the number of pulses and the applied electric field strength.

The results suggest that the effects of μsPEF exposure on AF cells depend on both the number of pulses and the applied electric field strength. Specifically, the observed earlier loss in cell viability at 24 h after μsPEF exposures is mainly attributed to the number of pulses. This is consistent with previous studies that have shown that high numbers of pulses can cause irreversible damage to the cell membrane, leading to cell death.^42,43^ When the number of pulses is high, the cumulative effect can lead to extensive membrane damage and cell death.

**Figure 3:**
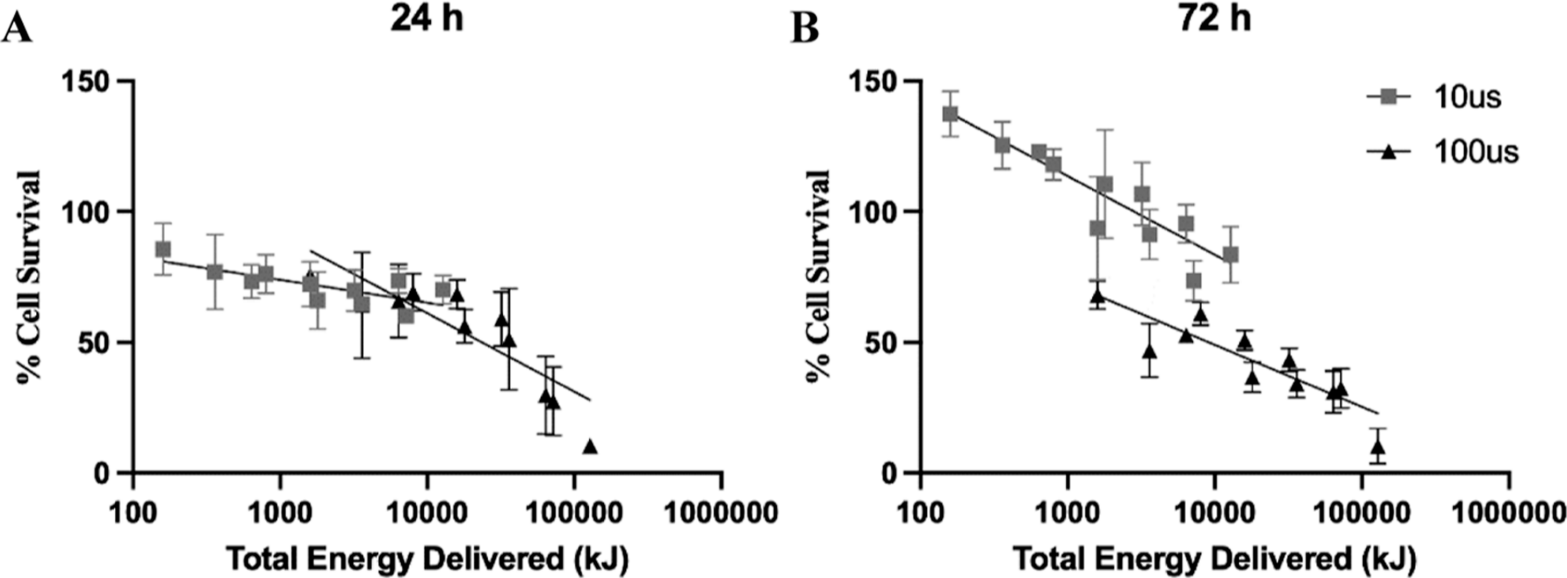
Semilog plots of mean values of AF cell viability with respect to TED from exposures to 10 and 100 μsPEF. (A) AF cell viability at 24 h postexposure. (B) AF cell viability at 72 h postexposure. Cell viability exceeds 100% of the controls’ mean values, and hence, the *y*-axes go up to 150%. Data are from *n* = 5 independent experiments, and error bars represent 1 SD.

Plots of migration rate versus total energy delivered (TED) show semilogarithmic relationships for 10 and 100 μsPEF exposures (Figure 3). These TED plots enable direct comparison of the effect of pulse duration on cell viability at 24 and 72 h after exposure. After 24 h, a biphasic relationship appears between pulse duration and TED, with a noticeably gradual downward slope associated with 10 μsPEF and a steeper slope with 100 μs PEF (Figure 3A). After 72 h, the slopes are similar for 10 and 100 μsPEF, but cell viabilities following 10 μsPEF exposure are offset approximately 30% above those following 100 μsPEF exposure for a given TED value (Figure 3B).

### 2.2 Effect of μsPEF Exposures on AF Cell Migration

**Figure 4:**
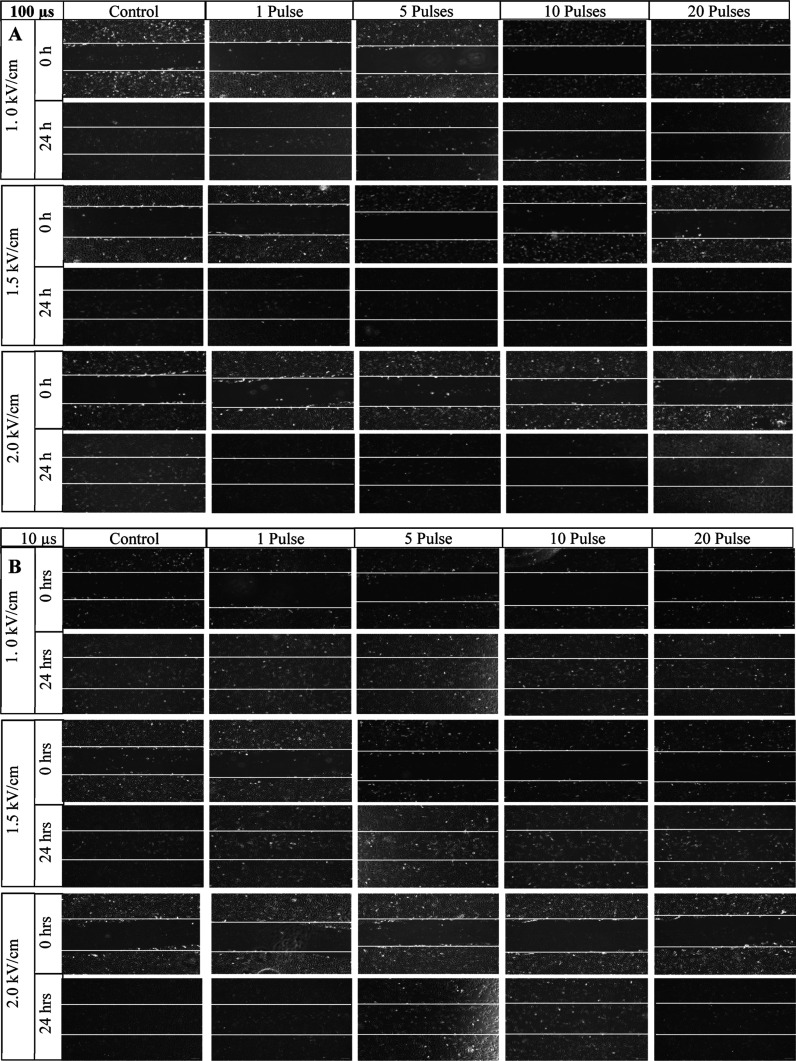
Representative phase microscopy images of AF cell migration at 0 and 24 h after (A) 100 μs and (B) 10 μsPEF exposure. Scale bar: 100 μm.

**Figure 5:**
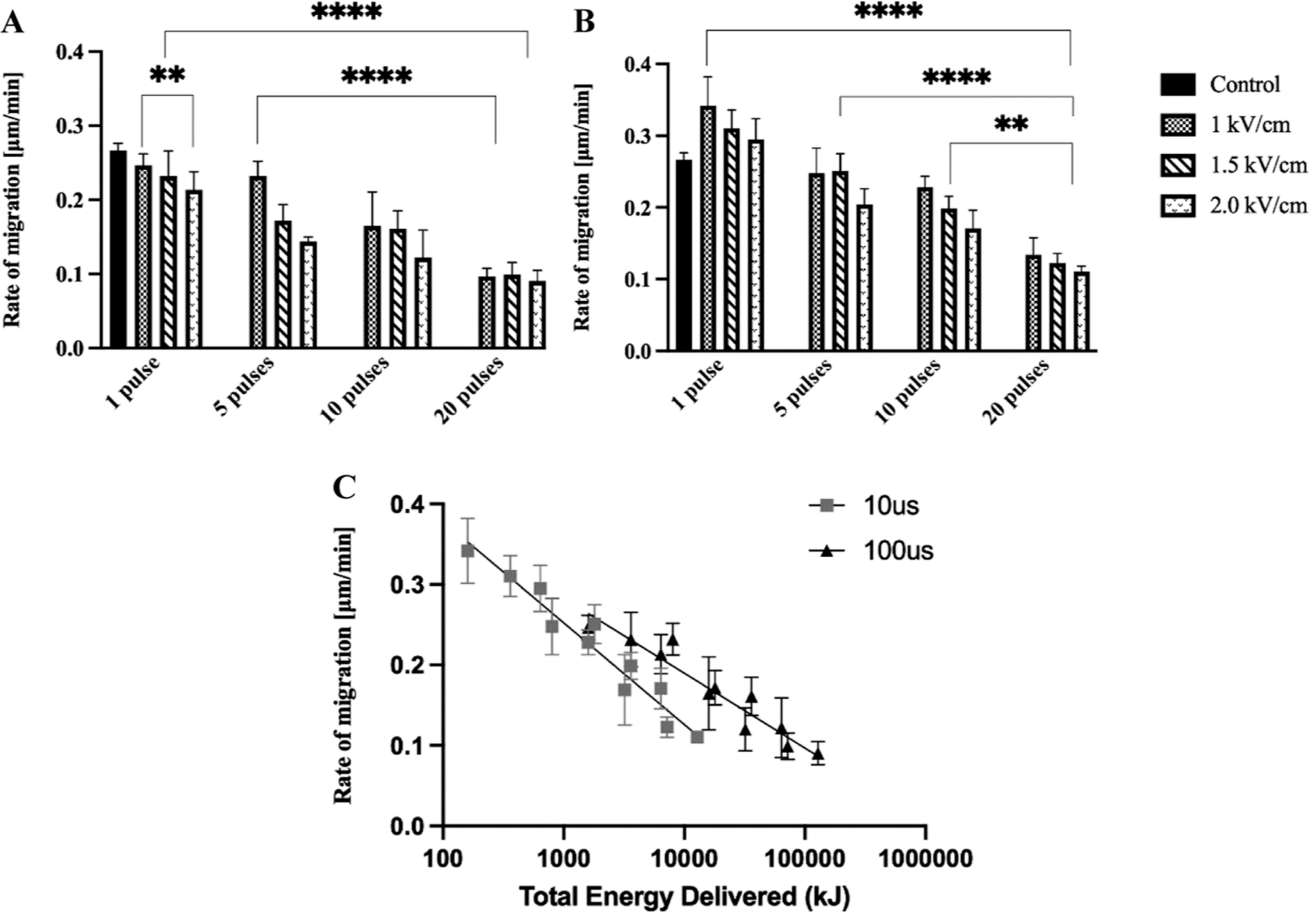
Rate of migration of AF cells exposed to (A) 100 μsPEF and (B) 10 μsPEF after 24 h. (C) Rate of migration versus TED as a function of pulse duration, with lines representing a semilogarithmic fit. Results are presented as mean ± SD; **p < 0.01, ***p < 0.001, and ****p < 0.0001.

To study the effects of μsPEF exposure on AF cell migration, a manual wound of ∼400 μm gap width has been formed within the AF cell monolayer, which is then subjected to a train of μsPEF. The cells are monitored using an inverted phase microscope for imaging every 3 h. Figures 4 and 5 compare the wound model before and 24 h after μsPEF exposures to 100 and 10 μs pulses, respectively. The average wound width along the scratch is measured to determine the average rate of migration of the cells after μsPEF exposure. The average rate of migration with respect to time is plotted for cells exposed to 1, 5, 10, and 20 pulses at 1.0, 1.5, and 2.0 kV/cm for both 10 and 100 μsPEF in Figure 5. The average rate of migration of the unexposed AF cells exceeds the rate of migration of the exposed cells. However, the rate of migration is enhanced in cells exposed to 1 pulse at 1.0 kV/cm of 10 μsPEF. The rate of migration is generally affected by the number of pulses as well as the applied electric field strength for both pulse widths. For 10 and 100 μsPEF exposures, the average rate of migration decreases with increasing number of pulses. The relation between the rate of migration and number of pulses is emphasized by an average *p*-value < 0.001 for both 10 and 100 μsPEF exposure (Figure 5A,B). Plots of migration rate versus TED also reveal semilogarithmic relationships for both 10 μsPEF (*R*^2^ = 0.837) and 100 μsPEF (*R*^2^ = 0.766) exposures (Figure 5C). According to these model fits, the average migration rates (*C*_r_) of AF cells exposed to 10 and 100 μsPEF are, respectively,
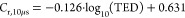
1
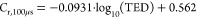
2

The 10 μs pulse width exposures exhibit higher rates of cell migration after exposure compared to the cells treated with 100 μs pulse width exposures. This observation correlates with the reduced cell viability with 100 μs of PEF exposures. The apparent dislodging of adhered cells after exposure to 10 and 20 pulses of the 100 μs pulse width also is noteworthy, as it suggests that μsPEF exposure could have significant effects on cell adhesion and morphology.

## 3 Discussion

Microsecond- and nanosecond-duration PEF exposures above a threshold electric field strength induce important changes in cell physiology by permeabilizing the cell membrane.^11^ μsPEFs are effectively used in several research areas such as medicine and biotechnology—for antitumor electrochemotherapy, tumor ablation, cell transfection, etc. PEF exposures induce transmembrane potential changes that can create electropores within the biological membranes. μsPEF interacts with the plasma membrane and changes its permeability properties once the field amplitude reaches a certain threshold. The electrical parameters selected for the μsPEF stimulation herein cause AF electropermeabilization.

Our experimental results demonstrate that the varied μsPEF parameters (number of pulses, applied field strength, and pulse duration) differentially influence the lethal and stimulating effects of exposure on primary bovine AF fibroblast-like cells. The MTT viability assay at 24 and 72 h revealed cell death and proliferation. These parametric relationships are complex for AF cells. The 10 μsPEF treatment causes cell death within the sample populations, but cell viability and proliferation have increased within 72 h postexposure. The effect of the number of pulses is more significant for the 100 μsPEF treatments, and the applied electric field strength has a greater contribution to the observed cell responses than that for the 10 μsPEF treatments. Therefore, a 10 μs pulse width generally induces reversible electroporation, whereas the 100 μs pulse width induces irreversible electroporation in the primary bovine AF fibroblast-like cells.

The divergent cellular responses to 10 and 100 μs of PEF exposures are not unexpected. Sankaranarayanan et al. report irreversible electroporation in chick embryo fibroblast cells exposed to 1 and 8 pulses at 1.2 kV/cm with 100 μsPEF.^44^ Hanna et al. show reduced cell viability in the Chinese hamster DC-3F cell line after exposure to 1 pulse of 100 μsPEF at 1.4–2.0 kV/cm, although they show that human amniotic mesenchymal stromal stem cell (haMSC) mortality is not reduced after exposure to a similar, single 100 μsPEF at 2.0 kV/cm.^14^ Thus, different cell types are expected to respond differently to a given pulse parameter set of μsPEF exposure.

The application of PEF has also been reported to enhance proliferation in other cell types. Dubey et al. report increased proliferation of mouse fibroblast L929 cells after exposure to direct current (DC) at 15 V.^8^ Primary mouse muscle myoblast and human vertebrae osteoblast cells have also been shown to proliferate at a rate up to 5-fold greater when exposed to 10–20 pulses of 300 nsPEF at 2.5–10 kV/cm.^9^ Hartig et al. show that proliferation of primary osteoblast-like bovine periosteum cells increases after exposure to 125 ms of sawtooth PEF at 0.06 kV/cm.^24^ Also, Fitzsimmons et al. use asymmetric biphasic PEF exposures of 15 pulses of 230 μs duration at 10 mV/cm to enhance the proliferation of normal human chondrocytes by up to 150%.^45^ Generally, our findings agree with what is reported for nsPEF and msPEF exposures—empowered proliferation is obtained with lower intensity pulse parameters.

Based on the analysis of the rate at which the wound gap closed, bovine AF cells exposed to a single pulse of 10 μsPEF at 1.0 kV/cm increased in migration speed compared to the unexposed control group. However, bovine AF cells exposed to up to 20 pulses at up to 2.0 kV/cm of 10 μsPEF expressed a dramatic decrease in migration speed compared to the control (Figure 5B). On the other hand, fibroblast cells exposed to 100 μsPEF have a significantly reduced rate of cell migration compared to control samples and to the samples exposed to 10 μsPEF (Figure 5A). The differential impacts of 10 and 100 μsPEF on the migration rate are highlighted via calculation of TED, which accounts for variation of both the number of pulses and electric field strength, allowing for more direct comparison of the effects of pulse duration. Not only can a 10 μsPEF exposure increase average AF cell migration rates over unexposed cells but also a given migration rate is achieved with less TED input from a 10 μsPEF than from a 100 μsPEF exposure (Figure 5C). μsPEF appears to change the morphology of the bovine AF cells, influencing the cell migration behavior and rate, depending on the selected PEF parameters. This is not unprecedented. Xiang et al. report increased fibroblast cell migration speed with a single 100 μsPEF at 0.75 kV/cm stimulation, whereas fibroblasts exposed to 1.5 kV/cm stimulation express a remarkable decrease in migration speed.^46^ When pulse parameters are selected such that a higher cell viability is maintained, μsPEF stimulation could promote an increased migration speed of primary AF cells over a long period, which has the potential to significantly reduce clinical treatment time and bolster regenerative tissue engineering.

The mechanisms by which μsPEF exposure affects cells are complex and multifaceted. The enhanced proliferation and mechanisms of reduced cell viability observed at 72 h may involve activation of intracellular signaling pathways,^47^ while earlier reductions in cell viability at 24 h involves irreversible membrane damage and disruption of intracellular components and homeostasis. Further investigations of the specific mechanisms underlying the effects of μsPEF exposures on AF cells and optimization of the parameters for specific applications are needed. For instance, if the goal is to induce cell death, then high numbers of pulses can be used. On the other hand, if the goal is to enhance cell proliferation and migration, a combination of a small number of shorter duration μsPEFs below a critical threshold of applied electric field strength can be used to optimize the desired effects. Following this first report of enhanced cell migration and proliferation induced by μsPEF exposure of AF cells, further investigations should determine pulse parameter spaces that elicit similar responses in other types of cells, e.g., Chinese hamster ovary cells and human stem cells, that would impact healthcare and life sciences industries.

## 4 Materials and Methods

### 4.1 Fibroblast Cell Isolation and Culture Conditions

Whole bovine tails were acquired from a local abattoir (Bringhurst Meets, Berlin, NJ, USA) without the skin to reduce contamination. Intact IVDs are dissected using #10 and #22 scalpels within 4 h of sacrifice while being kept hydrated in saline. Under aseptic conditions, AF tissues were isolated with a biopsy punch (6 mm diameter) and transferred to a specimen container. The isolated fibrocartilage tissue was minced and incubated overnight in collagenase P solution, made by dissolving 50 μg of collagenase enzyme (Rockland Immunochemicals, Philadelphia, PA, USA) in 10 mL of growth media. The growth media consisted of Dulbecco’s modified Eagle’s Medium (Thermo Fisher Scientific, Bridgewater, NJ, USA) supplemented with 10% fetal bovine serum (Thermo Fisher Scientific, Bridgewater, NJ, USA) and 1% antibiotic–antimycotic acid (Thermo Fisher Scientific, Bridgewater, NJ, USA). After digestion, the suspension was sieved through a 100 μm cell strainer and centrifuged at 1200 rpm for 5 min, and the pellet was resuspended in fresh growth media. The cells were counted and seeded in monolayer cell culture flasks at a concentration of 2 × 10^5^ cells/mL and then incubated at 95% humidity, 5% CO_2_, and 37 °C. At 90% confluency, the cells were trypsinized and resuspended in media for PEF exposures.

### 4.2 μsPEF Exposures

The PEF exposure system used to execute the pulses was a commercially available pulsing system consisting of a pulse generator (BTX Gemini X2), a cuvette holder, standard 2 mm gap aluminum electrode electroporation cuvettes (Bulldog Bio, Portsmouth, NH, USA), and a gold-plated Petri Dish Pulser electrode array (BTX, Holliston, MA, USA). The cuvettes were used to expose cell suspensions for viability studies, whereas the Petri dish electrode was used to expose adhered cells for migration studies. The responses of primary AF cells to μsPEF were explored by exposing the cells to 10 and 100 μs pulse durations at different electric field strengths and pulse numbers. Rectangular pulse waveforms with 1, 5, 10, and 20 pulses at 1, 1.5, and 2 kV/cm were applied to the cells. Our COMSOL Multiphysics simulation of the applied fields was published.^42^ The sham controls for all the exposures were handled similarly, except pulses were not applied to the cells.

### 4.3 Viability Assay

For cell viability measurements using the MTT assay, cells were exposed at 2 × 10^6^ cells/mL in 2 mm gap electroporation cuvettes. Exposed cells were aliquoted into 96-well plates in triplicate at 2 × 10^4^ cells/well. The volume of the cell suspension was topped to 100 μL/well growth media and incubated at 37 °C and 5% CO_2_ for 24 or 72 h. After the appropriate time of incubation, 10 μL of MTT reagent, 3-(4,5-dimethylthiazol-2-yl)-2,5-diphenyltetrazolium bromide, was added to each well and further incubated for 4 h. The crystal dissolution solution was then used to dissolve the blue formazan crystals formed during incubation by adding 100 μL of the solvent to each well and shaking overnight on an orbital shaker. The absorbance was read at 570 nm using a Tecan Infinite F200pro microplate reader (Morrisville, NC, USA). The optical absorbance of the exposed cells was converted to cell density and normalized against the matched controls (unexposed cells). The total energy delivered (TED, in kJ) was calculated as a function of the media conductivity (σ = 1.6 S/m), electric field strength (*E*), pulse duration (*t*_p_), and number of pulses (*n*)
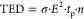
3

### 4.4 Migration Studies

At 90% confluency, the AF cells were trypsinized and seeded with growth media in a 60 mm^2^ Petri dish. The cells were then incubated in a humidified incubator at 37 °C and 5% CO_2_ until they reached 85–90% confluency. The media was aspirated, and the cells were washed with 2 mL of phosphate-buffered saline (PBS). A wound model was formed in the center of the plate by using the small end of a 10 μL polypropylene micropipette tip (Corning # 4115, Glendale, AZ, USA) to scratch a cell-free area (∼150 μm gap) into the confluent monolayer. The cells were rinsed twice with 1 mL of PBS to remove all debris. Complete growth media was added to the cells, and images were taken using an Olympus CKX53 inverted phase microscope using a 10×, 0.25 NA objective (Tokyo, Japan). The cells were then exposed to μsPEF using a Petri Dish Pulser electrode array. The electrodes were placed 1 mm above the cell monolayer during pulse exposures. Phase microscopy images were taken at 3 h intervals for a total of 24 h duration for migration distance analysis. Four fiduciary markings made with a permanent marker on the bottom of the Petri dish glass ensured consistent positioning for image acquisition. Images were analyzed for gap measurements using Fiji (a distribution of ImageJ2).^48^

### 4.5 Statistical Analysis

Statistical analysis of all data was performed in GraphPad Prism 9 (San Diego, CA, USA) using two-way ANOVA with Tukey’s post hoc test for multiple comparisons. A confidence interval of 95% was applied for all data analyses. The error values were reported as 1 SD of the arithmetic mean.

## References

[ref1] Roberts S.; Evans H.; Trivedi J.; Menage J. Histology and pathology of the human intervertebral disc. J. Bone Joint Surg. 2006, 88 (Suppl 2), 10–14. 10.2106/jbjs.F.00019. 16595436

[ref2] Jin L.; Liu Q.; Scott P.; Zhang D.; Shen F.; Balian G.; Li X. Annulus fibrosus cell characteristics are a potential source of intervertebral disc pathogenesis. PLoS One 2014, 9 (5), e96519 10.1371/journal.pone.0096519. 24796761 PMC4010482

[ref3] Sakai D.; Schol J. Cell therapy for intervertebral disc repair: Clinical perspective. J. Orthop. Translat. 2017, 9, 8–18. 10.1016/j.jot.2017.02.002. 29662795 PMC5822958

[ref4] Mern D. S.; Beierfuß A.; Thomé C.; Hegewald A. A. Enhancing human nucleus pulposus cells for biological treatment approaches of degenerative intervertebral disc diseases: a systematic review. J. Tissue Eng. Regener. Med. 2014, 8 (12), 925–936. 10.1002/term.1583. 22927290

[ref5] Benneker L. M.; Andersson G.; Iatridis J. C.; Sakai D.; Härtl R.; Ito K.; Grad S. Cell therapy for intervertebral disc repair: advancing cell therapy from bench to clinics. Eur. Cell. Mater. 2014, 27, 5–11. 10.22203/ecm.v027sa02. 24802611 PMC5072777

[ref6] Hu B.; He R.; Ma K.; Wang Z.; Cui M.; Hu H.; Rai S.; Wang B.; Shao Z. Intervertebral Disc-Derived Stem/Progenitor Cells as a Promising Cell Source for Intervertebral Disc Regeneration. Stem Cell. Int. 2018, 2018, 7412304 10.1155/2018/7412304. PMC6312624 30662469

[ref7] Sakai D.; Andersson G. B. Stem cell therapy for intervertebral disc regeneration: obstacles and solutions. Nat. Rev. Rheumatol. 2015, 11 (4), 243–256. 10.1038/nrrheum.2015.13. 25708497

[ref8] Dubey A. K.; Gupta S. D.; Basu B. Optimization of electrical stimulation parameters for enhanced cell proliferation on biomaterial surfaces. J. Biomed. Mater. Res. B Appl. Biomater. 2011, 98 (1), 18–29. 10.1002/jbm.b.31827. 21432997

[ref9] Vadlamani R. A.; Nie Y.; Detwiler D. A.; Dhanabal A.; Kraft A. M.; Kuang S.; Gavin T. P.; Garner A. L. Nanosecond pulsed electric field induced proliferation and differentiation of osteoblasts and myoblasts. J. R. Soc., Interface 2019, 16 (155), 20190079 10.1098/rsif.2019.0079. 31213169 PMC6597781

[ref10] Pakhomov A. G.; Shevin R.; White J. A.; Kolb J. F.; Pakhomova O. N.; Joshi R. P.; Schoenbach K. H. Membrane permeabilization and cell damage by ultrashort electric field shocks. Arch. Biochem. Biophys. 2007, 465 (1), 109–118. 10.1016/j.abb.2007.05.003. 17555703

[ref11] Breton M.; Mir L. M. Microsecond and nanosecond electric pulses in cancer treatments. Bioelectromagnetics 2012, 33 (2), 106–123. 10.1002/bem.20692. 21812011

[ref12] Cemazar M.; Jarm T.; Miklavcic D.; Macek A.; Alojz I.; Kopitar A. N.; Sersa G. Effect of electric-field intensity on electropermeabilization and electrosensitivity of various tumor-cell lines in vitro. Electro- Magnetobiol. 1998, 17, 263–272. 10.3109/15368379809022571.

[ref13] Saulis G.; Saulė R.; Bitinaitė A.; Žurauskienė N.; Stankevič V.; Balevičius S. Theoretical Analysis and Experimental Determination of the Relationships Between the Parameters of the Electric Field Pulse Required to Electroporate the Cells. IEEE Trans. Plasma Sci. 2013, 41 (10), 2913–2919. 10.1109/TPS.2013.2276918.

[ref14] Hanna H.; Denzi A.; Liberti M.; André F. M.; Mir L. M. Electropermeabilization of Inner and Outer Cell Membranes with Microsecond Pulsed Electric Fields: Quantitative Study with Calcium Ions. Sci. Rep. 2017, 7 (1), 13079 10.1038/s41598-017-12960-w. 29026094 PMC5638809

[ref15] Neumann E.; Sowers A. E.; Jordan C. A. Electroporation and Electrofusion in Cell Biology; Springer: New York, 1989.

[ref16] Choromańska A.; Chwiłkowska A.; Kulbacka J.; Baczyńska D.; Rembiałkowska N.; Szewczyk A.; Michel O.; Gajewska-Naryniecka A.; Przystupski D.; Saczko J. Modifications of Plasma Membrane Organization in Cancer Cells for Targeted Therapy. Molecules 2021, 26 (7), 1850 10.3390/molecules26071850. 33806009 PMC8037978

[ref17] Wang Y.; Jiang T.; Xie L.; Wang H.; Zhao J.; Xu L.; Fang C. Effect of pulsed field ablation on solid tumor cells and microenvironment. Front. Oncol. 2022, 12, 899722 10.3389/fonc.2022.899722. 36081554 PMC9447365

[ref18] Garner A. L.; Neculaes V. B.; Deminsky M.; Dylov D. V.; Joo C.; Loghin E. R.; Yazdanfar S.; Conway K. R. Plasma membrane temperature gradients and multiple cell permeabilization induced by low peak power density femtosecond lasers. Biochem. Biophys. Rep. 2016, 5, 168–174. 10.1016/j.bbrep.2015.11.019. 28955820 PMC5598230

[ref19] Garner A. L.; Chen N.; Yang J.; Kolb J.; Swanson R. J.; Loftin K. C.; Beebe S. J.; Joshi R. P.; Schoenbach K. H. Time domain dielectric spectroscopy measurements of HL-60 cell suspensions after microsecond and nanosecond electrical pulses. IEEE Trans. Plasma Sci. 2004, 32, 2073–2084. 10.1109/TPS.2004.835973.

[ref20] Casciati A.; Tanori M.; Gianlorenzi I.; Rampazzo E.; Persano L.; Viola G.; Cani A.; Bresolin S.; Marino C.; Mancuso M.; et al. Effects of Ultra-Short Pulsed Electric Field Exposure on Glioblastoma Cells. Int. J. Mol. Sci. 2022, 23 (6), 3001 10.3390/ijms23063001. 35328420 PMC8950115

[ref21] Ibey B. L.; Pakhomov A. G.; Gregory B. W.; Khorokhorina V. A.; Roth C. C.; Rassokhin M. A.; Bernhard J. A.; Wilmink G. J.; Pakhomova O. N. Selective cytotoxicity of intense nanosecond-duration electric pulses in mammalian cells. Biochim. Biophys. Acta 2010, 1800 (11), 1210–1219. 10.1016/j.bbagen.2010.07.008. 20691249 PMC2934740

[ref22] May R. D.; Tekari A.; Frauchiger D. A.; Krismer A.; Benneker L. M.; Gantenbein B. Efficient Nonviral Transfection of Primary Intervertebral Disc Cells by Electroporation for Tissue Engineering Application. Tissue Eng., Part C 2017, 23 (1), 30–37. 10.1089/ten.tec.2016.0355. 27968705

[ref23] Atsu P. M.; Thompson G. L. Electrical impedance decreases in annulus fibrosus cartilage exposed to microsecond pulsed electric fieldsex vivo. Biomed. Phys. Eng. Express 2023, 9 (2), 025017 10.1088/2057-1976/acbd54. 36806548

[ref24] Hartig M.; Joos U.; Wiesmann H. P. Capacitively coupled electric fields accelerate proliferation of osteoblast-like primary cells and increase bone extracellular matrix formation in vitro. Eur. Biophys. J. 2000, 29 (7), 499–506. 10.1007/s002490000100. 11156291

[ref25] Brighton C. T.; Jensen L.; Pollack S. R.; Tolin B. S.; Clark C. C. Proliferative and synthetic response of bovine growth plate chondrocytes to various capacitively coupled electrical fields. J. Orthop. Res. 1989, 7 (5), 759–765. 10.1002/jor.1100070519. 2760750

[ref26] Buchmann L.; Frey W.; Gusbeth C.; Ravaynia P. S.; Mathys A. Effect of nanosecond pulsed electric field treatment on cell proliferation of microalgae. Bioresour. Technol. 2019, 271, 402–408. 10.1016/j.biortech.2018.09.124. 30296747

[ref27] Guney Eskiler G. The Interaction of PI3K Inhibition with Homologous Recombination Repair in Triple Negative Breast Cancer Cells. J. Pharm. Pharm. Sci. 2019, 22 (1), 599–611. 10.18433/jpps30684. 31804921

[ref28] Gu H.; Maeda H.; Moon J. J.; Lord J. D.; Yoakim M.; Nelson B. H.; Neel B. G. New role for Shc in activation of the phosphatidylinositol 3-kinase/Akt pathway. Mol. Cell. Biol. 2000, 20 (19), 7109–7120. 10.1128/MCB.20.19.7109-7120.2000. 10982827 PMC86258

[ref29] Miao D.; Zhang L. Leptin modulates the expression of catabolic genes in rat nucleus pulposus cells through the mitogen-activated protein kinase and Janus kinase 2/signal transducer and activator of transcription 3 pathways. Mol. Med. Rep. 2015, 12 (2), 1761–1768. 10.3892/mmr.2015.3646. 25892402 PMC4464091

[ref30] Lin Y.; Wang F.; Zhang G. L. Natural products and their derivatives regulating the janus kinase/signal transducer and activator of transcription pathway. J. Asian Nat. Prod. Res. 2014, 16 (7), 800–812. 10.1080/10286020.2014.929573. 25076196

[ref31] Yuan X.; Arkonac D. E.; Chao P. H.; Vunjak-Novakovic G. Electrical stimulation enhances cell migration and integrative repair in the meniscus. Sci. Rep. 2014, 4, 3674 10.1038/srep03674. 24419206 PMC3891019

[ref32] Jezierska-Woźniak K.; Lipiński S.; Grabarczyk Ł.; Barczewska M.; Habich A.; Wojtkiewicz J.; Maksymowicz W. Migration of human mesenchymal stem cells stimulated with pulsed electric field and the dynamics of the cell surface glycosylation. Adv. Clin. Exp. Med. 2018, 27 (9), 1181–1193. 10.17219/acem/90872. 29963783

[ref33] Tai G.; Tai M.; Zhao M. Electrically stimulated cell migration and its contribution to wound healing. Burns Trauma 2018, 6, 20 10.1186/s41038-018-0123-2. 30003115 PMC6036678

[ref34] Funk R. H. Endogenous electric fields as guiding cue for cell migration. Front. Physiol. 2015, 6, 143 10.3389/fphys.2015.00143. 26029113 PMC4429568

[ref35] Chang K.; Chang W. H.; Wu M. L.; Shih C. Effects of different intensities of extremely low frequency pulsed electromagnetic fields on formation of osteoclast-like cells. Bioelectromagnetics 2003, 24 (6), 431–439. 10.1002/bem.10118. 12929162

[ref36] Aaron R. K.; Boyan B. D.; Ciombor D. M.; Schwartz Z.; Simon B. J. Stimulation of growth factor synthesis by electric and electromagnetic fields. Clin. Orthop. Relat. Res. 2004, 419 (419), 30–37. 10.1097/00003086-200402000-00006. 15021128

[ref37] Ren X.; Levin D.; Lin F. Cell Migration Research Based on Organ-on-Chip-Related Approaches. Micromachines 2017, 8 (11), 324 10.3390/mi8110324. 30400514 PMC6190356

[ref38] Colvin R. A.; Means T. K.; Diefenbach T. J.; Moita L. F.; Friday R. P.; Sever S.; Campanella G. S.; Abrazinski T.; Manice L. A.; Moita C.; et al. Synaptotagmin-mediated vesicle fusion regulates cell migration. Nat. Immunol. 2010, 11 (6), 495–502. 10.1038/ni.1878. 20473299 PMC2951881

[ref39] Gao R. C.; Zhang X. D.; Sun Y. H.; Kamimura Y.; Mogilner A.; Devreotes P. N.; Zhao M. Different roles of membrane potentials in electrotaxis and chemotaxis of dictyostelium cells. Eukaryot. Cell 2011, 10 (9), 1251–1256. 10.1128/EC.05066-11. 21743003 PMC3187056

[ref40] Krishnamoorthy S.; Zhang Z.; Xu C. Guided cell migration on a graded micropillar substrate. Bio-Des. Manuf. 2020, 3 (1), 60–70. 10.1007/s42242-020-00059-7.

[ref41] Jamil M. M. A.; Zaltum M. A. M.; Youseffi M.; Javid F. Study on Pulse Electric Field Exposure Effect on HeLa Cells For Wound Healing Application. J. Phys.: Conf. Ser. 2019, 1372 (1), 012021 10.1088/1742-6596/1372/1/012021.

[ref42] Jiang C.; Davalos R. V.; Bischof J. C. A review of basic to clinical studies of irreversible electroporation therapy. IEEE Trans. Biomed. Eng. 2015, 62 (1), 4–20. 10.1109/TBME.2014.2367543. 25389236

[ref43] Aycock K. N.; Davalos R. V. Irreversible electroporation: Background, theory, and review of recent development in clinical oncology. Bioelectricity 2019, 1 (4), 214–234. 10.1089/bioe.2019.0029. 34471825 PMC8370296

[ref44] Sundararajan R.; Rajendran R.; Shahid S. S.; Santosh D. K.; Radhakrishnan S.; Priyadarshan K.; Varsha S.; Kumar U. V.; Ramachandran R.; Sankaranarayanan K. Effect of irreversible electroporation on cancer cells. 2011 Annual Report Conference on Electrical Insulation and Dielectric Phenomena, 2011; pp 164–167.

[ref45] Fitzsimmons R. J.; Gordon S. L.; Kronberg J.; Ganey T.; Pilla A. A. A pulsing electric field (PEF) increases human chondrocyte proliferation through a transduction pathway involving nitric oxide signaling. J. Orthop. Res. 2008, 26 (6), 854–859. 10.1002/jor.20590. 18240331

[ref46] Xiang X. W.; Liu H. T.; Liu W.; Yan Z. Y.; Zeng Y. L.; Wang Y. J.; Liu J.; Chen Y. C.; Yu S. X.; Zhu C. H.; Microsecond pulse electrical stimulation modulates cell migration. 2022. bioRxiv 2022.10.23.513372.

[ref47] Safaei Z.; Thompson G. L. Histone deacetylase 4 and 5 translocation elicited by microsecond pulsed electric field exposure is mediated by kinase activity. Front. Bioeng. Biotechnol. 2022, 10, 10.3389/fbioe.2022.1047851. PMC9713944 36466344

[ref48] Schindelin J.; Arganda-Carreras I.; Frise E.; Kaynig V.; Longair M.; Pietzsch T. Fiji: An open-source platform for biological-image analysis. Nat. Methods 2012, 9 (7), 676–682. 10.1038/nmeth.2019. 22743772 PMC3855844

